# *Commiphora myrrha* extract protects pigeons from *Eimeria labbeana*-like-triggered inflammatory dysregulation

**DOI:** 10.3389/fimmu.2025.1714313

**Published:** 2025-12-17

**Authors:** Rewaida Abdel-Gaber, Shurug Albasyouni, Simeon Santourlidis, Saleh Al Quraishy, Esam Al-Shaebi

**Affiliations:** 1 Department of Zoology, College of Science, King Saud University, Riyadh, Saudi Arabia; 2 Epigenetics Core Laboratory, Institute of Transplantation Diagnostics and Cell Therapeutics, Medical Faculty, Heinrich-Heine University Düsseldorf, Düsseldorf, Germany

**Keywords:** coccidiosis, *Commiphora myrrha*, immune modulation, inflammatory dysregulation, natural agents, pigeons

## Abstract

**Background:**

Coccidiosis, caused by *Eimeria* species, is a major enteric disease in birds, with *Eimeria labbeana*-like isolates frequently inducing severe intestinal lesions, diarrhea, and reduced weight gain in pigeons. Conventional anticoccidial drugs face limitations due to resistance, residue concerns, and environmental impact, highlighting the need for alternative strategies. *Commiphora myrrha* (myrrh) is a resinous plant extract rich in bioactive compounds with antioxidant, antimicrobial, antiparasitic, and anti-inflammatory properties. This study evaluated the protective effects of *C. myrrha* resin in pigeons experimentally infected with *E. labbeana*-like isolates.

**Methods:**

Resin of *C. myrrha* was collected from Riyadh, Saudi Arabia, authenticated, and extracted with 70% methanol to prepare a crude extract (MyE). Its chemical composition was characterized using GC–MS. A laboratory strain of *Eimeria labbeana*-like oocysts was propagated in pigeons, sporulated, and used for experimental infection. Twenty-five pigeons were randomly assigned to five groups: uninfected control, uninfected + myrrh extract (MyE), infected control, infected + MyE, and infected + amprolium (standard drug). MyE and amprolium treatments were administered orally for 5 days post-infection. Parasitological, histological, immunohistochemical (NF-κB and IFN-γ), gene expression (MUC2, IL-1β, IL-10, IFN-γ, and TNF-α), and cytokine (IL-10 and TNF-α) analyses were conducted.

**Results:**

In this study, myrrh resin was methanol-extracted and characterized by GC–MS, revealing 29 phytochemical components. Experimental infection of pigeons with *E. labbeana*-like oocysts resulted in peak fecal oocyst shedding (~5.25 × 10^5^ oocysts/g.feces), extensive development of intracellular parasite stages (meronts, gamonts, and developing oocysts), a marked reduction in goblet cell numbers, and elevated intestinal inflammatory responses, including increased NF-κB and IFN-γ immunoreactivity, as well as upregulated mRNA expression of IL-1β, IL-10, IFN-γ, and TNF-α. Oral administration of MyE significantly suppressed oocyst shedding by 60%, reduced the number of intracellular parasitic stages, restored goblet cell counts, and downregulated both gene and protein levels of pro-inflammatory markers while enhancing MUC2 expression, indicating effective modulation of *Eimeria*-induced intestinal damage and inflammatory dysregulation.

**Conclusion:**

These findings demonstrate that *C. myrrha* extract effectively mitigates *Eimeria*-induced intestinal damage, inflammation, and immune dysregulation, highlighting its potential as a natural, plant-based intervention for managing pigeon coccidiosis.

## Introduction

Coccidiosis is a major enteric disease in birds, primarily caused by protozoan parasites of the genus *Eimeria* (1–4). All *Eimeria* species exhibit a complex life cycle with schizogony, gametogony, and sporogony phases (5). The infectious forms are fully sporulated oocysts, which invade the gastrointestinal mucosa, leading to intestinal lesions, dehydration, and bloody diarrhea (6, 7). Disease progression triggers inflammatory and oxidative responses that exacerbate tissue damage and impair bird performance (8). In pigeons, *Eimeria labbeana* (or *E. labbeana*-like isolates) is frequently associated with severe intestinal pathology and weight loss (2, 8–11). These infections also increase susceptibility to secondary pathogens, further compromising bird health (7, 12, 13).

Traditional management of avian coccidiosis relies heavily on synthetic anticoccidial drugs and ionophores (5). However, long-term use has contributed to the emergence of resistant *Eimeria* strains, and concerns remain regarding drug residues in animal products, environmental impacts, and consumer demand for residue-free meat and eggs (14–16). These limitations highlight the need to evaluate alternative or complementary approaches. Nutritional strategies, improved management practices, and phytotherapeutics have shown promise in supporting gut health, enhancing immunity, and reducing dependence on conventional chemotherapeutics (16–18). While anticoccidial drugs remain widely used as reference treatments in experimental studies, their effectiveness may be compromised by resistance, and their practical utility in long-term, sustainable poultry management is limited.

*Commiphora myrrha*, commonly known as myrrh, is a resinous substance obtained from the bark of trees native to Africa, the Middle East, and Asia (19). Its therapeutic properties have been recognized in traditional medicine for centuries (20). Phytochemical studies reveal that myrrh contains terpenoids, sesquiterpenes, and other bioactive secondary metabolites, which exhibit antioxidant, antimicrobial, antiparasitic, and anti-inflammatory effects (21, 22). These properties suggest myrrh could modulate the inflammatory and oxidative pathways activated by *Eimeria* infection, potentially limiting tissue damage and restoring physiological balance. Despite growing interest in its bioactivities, experimental evidence of myrrh’s efficacy against avian coccidiosis, particularly *E. labbeana*-like infections in pigeons, remains limited. Recent studies have highlighted its antimicrobial and immunomodulatory potential in avian models, supporting its use as a natural prophylactic or adjunct therapy (23, 24).

This study investigates the potential of C. myrrha extract to protect pigeons against *E. labbeana*-like infection by (i) reducing intestinal inflammation, (ii) preserving mucosal architecture and barrier integrity, and (iii) modulating immune responses. By assessing parasitological, histopathological, biochemical, and immunological parameters, we aim to evaluate myrrh as a sustainable, plant-based alternative to conventional anticoccidial drugs, offering practical relevance in modern pigeon management.

## Materials and methods

### Plant collection and extract preparation

*Commiphora myrrha* (myrrh) resin was sourced from a local market in Riyadh, Saudi Arabia, and its identity was authenticated at the Botany Department Herbarium, King Saud University, where a voucher specimen was deposited (KSU-23539). For comparison, amprolium (AMP) (Amproxine 20%, water-soluble powder) was obtained from Gulf Veterinary Pharmacy, Riyadh, and used as a standard anticoccidial drug.

Following the method of Akande et al. (25), 100 g of resin was ground into a fine powder using a Hummer Grinder (Edison Electric, ED-CG1400, China). The powdered material was macerated in 1,000 mL of 70% methanol with gentle agitation at room temperature for 24 h. The extract was filtered through Whatman No. 1 filter paper, and the filtrate was concentrated using a Buchi rotary evaporator (Switzerland) at 45 °C. The resulting methanolic crude myrrh extract (MyE) was then reconstituted in distilled water (w/v) to prepare the required experimental doses.

### Gas chromatography-mass spectrometry analysis

Sample analysis was performed using an Agilent Technologies 7890B GC-MS system (Santa Clara, CA, USA) equipped with an autosampler. A 1 µL aliquot of the extract was injected, and compounds were identified using the NIST-MS library database, following the method of Kanthal et al. (26). Separation was achieved on a DB-5 MS capillary column (Agilent Technologies). Helium served as the carrier gas at a constant flow rate of 1 mL/min. The injector temperature was set at 250 °C, operating in split mode (1:50). The oven program ranged from 50 °C to 250 °C, with a total run time of 61 min. Mass spectrometric conditions included an electron ionization source at 230 °C, a mass scan range of 40–500 g/mol, a scan rate of 1.56, and a solvent delay of 4 min.

#### Parasite strain and preparation

A laboratory strain of *Eimeria labbeana*-like, a representative coccidian parasite, was utilized in this experiment. The parasite used in this study was identified based on the morphological, morphometric, and molecular data previously established in our published work, *“*Morphology, morphometry, and phylogeny of the protozoan parasite, *Eimeria labbeana-like* (Apicomplexa, Eimeriidae), infecting *Columba livia domestica”.* To propagate the oocysts, five pigeons were used as hosts. Following the method described by Qudoos et al. (27), each bird was orally inoculated with 3×10^4^ sporulated oocysts. Shedding of oocysts was observed in the feces of infected pigeons on the eighth day post-inoculation. The recovered oocysts were subsequently induced to sporulate in 2.5% (w/v) potassium dichromate (K_2_Cr_2_O_7_) at 24 °C, as outlined by El-Ashram and Suo (28). The sporulated oocysts were then centrifuged in phosphate-buffered saline (PBS) at 2500 rpm for 5 minutes and rinsed three times with distilled water.

### Pigeons, housing, and experimental design

Twenty-five white domestic pigeons (*Columba livia domestica*), weighing between 300–380 g, were purchased from the local animal market in Riyadh, Saudi Arabia. The birds were acclimatized for one week under controlled conditions, including a 12-hour light/dark cycle and a temperature of 23 ± 5 °C, with free access to tap water and a balanced seed-based diet. They were maintained at the animal facility of the Department of Zoology, College of Science, King Saud University, Riyadh.

The pigeons were randomly assigned to five groups (five birds each) as follows:

- Group 1 (control): fed a basal diet with tap water only.
- Group 2 (non-infected treated): received MyE (500 mg/kg) without infection (29).
- Group 3 (infected control): inoculated with *E. labbeana*-like oocysts without treatment.
- Group 4 (infected + MyE): administered MyE (500 mg/kg) following infection with *E. labbeana*-like oocysts (29).
- Group 5 (infected + amprolium): treated with the anticoccidial drug amprolium (1 g soluble powder/L of water) after infection (29).

All birds in Groups 3–5 were orally inoculated with 3×10^4^ sporulated *E. labbeana*-like oocysts, as described by Qudoos et al. (27). Treatments with MyE and amprolium began on day 3 post-infection (p.i.) and continued daily for five consecutive days. On day 8 p.i., fresh fecal samples from the infected, untreated, and treated groups were collected. Oocyst counts were determined using flotation with saturated saline, followed by McMaster’s counting technique (30). Additionally, the suppression of oocyst shedding was calculated as follows:


$$\text{Oocyst suppression }(\%)=100-\frac{\text{Oocyst output in the treated group}}{\text{Oocyst output in the infected group}}\times 100$$

### Sample collection

At the end of the experiment, pigeons were humanely sacrificed in compliance with ethical guidelines, and the small intestine was carefully dissected. The collected samples were preserved in two ways: (i) portions of intestinal tissue were fixed in 10% neutral buffered formalin (NBF) for histological and immunohistochemical (IHC) analysis, and (ii) other portions were stored in RNA later^®^ solution (Qiagen) at –80 °C for subsequent mRNA and protein expression studies.

#### Histological examination and parasitic scoring

Intestinal samples were fixed in 10% neutral buffered formalin (NBF) for 24 hours, dehydrated, embedded in paraffin, and sectioned at a thickness of 5 µm, following the method of Adam and Caihak (31). Sections were stained with hematoxylin and eosin (H&E) to identify parasite stages in both infected and treated groups. In contrast, additional sections were stained with Alcian blue to assess goblet cells as described by Adam and Caihak (31). Microscopic examination and imaging were performed using an Olympus B×61 microscope (Tokyo, Japan), and parasite stages were quantified across ten well-oriented villous-crypt units (VCUs).

#### Immunohistochemical analysis

Intestinal tissues fixed in formalin were processed by dehydration in graded ethanol, cleared with xylene, embedded in paraffin, and sectioned at 5 µm thickness using a microtome. For IHC labeling, sections were deparaffinized, rehydrated, and treated with 3% H_2_O_2_ for 5 minutes to block endogenous peroxidase activity. They were then pre-incubated with a normal serum buffer (Diagnostic BioSystems, Serpentine, CA, USA) for 30 minutes, followed by incubation at 4 °C for 3 hours with primary antibodies against Nuclear Factor kappa B (NF-κB) and Interferon-gamma (IFN-γ) (Santa Cruz Biotechnology, CA, USA).

Subsequently, sections were treated with a biotinylated secondary antibody and streptavidin–horseradish peroxidase conjugate (Vision Biosystems Novocastra, UK) according to the kit protocol. Detection was carried out using 3,3′-diaminobenzidine (DAB) substrate (Vision Biosystems Novocastra), and counterstaining was performed with hematoxylin and eosin (Sigma Chemical Co.). Between each step, sections were rinsed with immunowash buffer (Vision Biosystems Novocastra). Finally, tissues were dehydrated in graded ethanol, cleared in xylene, and mounted with glass coverslips. All slides were examined for NF-κB and IFN-γ expression and photographed using an Olympus BX61 microscope (Tokyo, Japan).

#### RNA extraction and qRT-PCR

Total RNA was isolated from intestinal tissues using Trizol reagent (Invitrogen, USA). To remove potential genomic DNA contamination, samples were treated with DNase (Applied Biosystems, Darmstadt, Germany) for at least one hour. Complementary DNA (cDNA) was then synthesized using a reverse transcription kit (Qiagen, Hilden, Germany) following the manufacturer’s instructions.

Quantitative real-time PCR (qRT-PCR) was performed on an ABI Prism 7500HT detection system (Applied Biosystems, Darmstadt, Germany) using SYBR Green PCR master mix (Qiagen, Hilden, Germany). Gene expression analysis targeted MUC2 (goblet cell-related response), interleukin (IL)-1β, IL-10, interferon gamma (IFN-γ), and tumor necrosis factor alpha (TNF-α), with β-actin serving as the housekeeping reference gene. All primers were obtained from Qiagen (Hilden, Germany) (**Table 1**).

**Table 1:** Oligonucleotide primer sequences for reverse transcription PCR amplification in the experiment.

RNA target	Direction	Oligonucleotide sequence (5’-3’)	NCBI reference
MUC2	Forward	GAATGTGCCAAACAAAGC	XM_021283472.1
	Reverse	CTGATGTACGCAAACCCT	
IL-1β	Forward	AAGTGCTTCGTGCTGGAGTC	NM_001282824.1
	Reverse	ACGGTACAGAGCGATGTTGA	
IL-10	Forward	GCTCTGAACTGCTGGATGAA	XM_021298374.1
	Reverse	CTGGTGAAGGGTGCTGGT	
IFN-γ	Forward	TCTGACAAGTCAAAGGCGCA	NM_001282845.1
	Reverse	ATGCACAGCTTTGCGTTGAG	
TNF-α	Forward	GTGTTCAGTCCCTCCTCGTT	XM_005506079.2
	Reverse	TCAATCACAAGCAATGGGAGC	
β-actin	Forward	ATGTCGGTGATGAAGCCCAG	DQ022673.1
	Reverse	GGTGCCAGATCTTCTCCATGT	

The qRT-PCR amplification protocol consisted of an initial denaturation at 95°C for 10 min, followed by 40 cycles of 95°C for 15 s and 60°C for 60 s. A melting curve analysis was performed from 60°C to 95°C to verify the specificity of the amplified fragments. Amplification and data analysis were conducted using the Bio-Rad iMark Microplate Reader (SW 1.04.02.E). Relative gene expression levels were calculated using the comparative Ct method (2^−ΔΔCT^) as described by Livak and Schmittgen (32).

#### Sandwich enzyme-linked immunosorbent assay

Intestinal levels of IL-10 (MBS9364117) and TNF-α (MBS050796) were measured using commercial ELISA kits (MyBioSource, San Diego, CA, USA) following the manufacturer’s instructions. Briefly, standards of known concentrations were prepared to generate a standard curve ranging from 0 to 500 pg/ml. Samples and standards were added to the microplate wells and incubated as recommended. After washing to remove unbound material, a specific detection antibody was added, followed by a substrate solution. Optical density (OD) readings were measured at 450 nm using a Bio-Rad iMark Microplate Reader (SW 1.04.02.E). Cytokine concentrations in the samples were calculated from the standard curve and expressed in pg/ml.

### Statistical analysis

Data are expressed as mean ± standard deviation (SD). Group differences were evaluated using one-way analysis of variance (ANOVA) followed by Duncan’s multiple range test. Analyses were conducted using SPSS version 18 for Windows (SPSS Inc., Chicago, IL, USA). Statistical significance was indicated in figures using the symbols (*) and (#) at p ≤ 0.05. Detailed descriptions of these symbols are provided in the figure legends.

## Results

The analysis performed with GC-MS showed that the methanolic extract contains 29 phytochemical components that may be responsible for its activity (**Table 1**). The characterization of the extract’s components was carried out using their retention time (RT), molecular weight (MW), molecular formula (MF), and peak area with its percentage (%). These compounds are listed in **Table 2** according to their RTs.

**Table 2:** Identification of phytochemical compounds by GC-MS in MyE.

Retention time (RT) (min)	Proposed compound	Molecular weight (MW)	Peak area	Peak area percentage (%)	Molecular formula (MF)
20.796	4-Trifluoroacetoxytetradecane	310	348373	1.25	C_16_H_29_F_3_O_2_
21.174	Naphthalene, 1,2,3,5,6,8a-hexahydro-4,7-dimethyl-1-(1-methylethyl)-, (1S-cis)-	230	499817	1.80	C_15_H_24_
22.058	Cyclohexanemethanol, 4-ethenyl-α,α,4-trimethyl-3-(1-methylethenyl)-, [1R-(1α,3α,4β)]-	222	778134	2.80	C_15_H_26_O
23.373	Cycloisolongifolene, 8,9-dehydro-9-formyl-	230	1952297	7.01	C_16_H_22_O
23.931	(-)-Spathulenol	220	1827247	6.57	C_15_H_24_O
24.205	tau.-Cadinol	222	3920190	14.09	C_15_H_26_O
24.994	α-acorenol	222	573843	2.06	C_15_H_26_O
25.436	3,7-Cyclodecadien-1-one, 3,7-dimethyl-10-(1-methylethylidene)-, (E,E)-	218	834726	3.00	C_15_H_22_O
26.467	Isoaromadendrene epoxide	220	591568	2.13	C_15_H_24_O
27.950	2-Naphthalenemethanol, decahydro-α,α,4a-trimethyl-8-methylene-, [2R-(2α,4aα,8aβ)]-	222	161700	0.58	C_15_H_26_O
29.045	2H-Cycloheptafuran-2-one, 6-[1-(acetyloxy)-3-oxobutyl]-3,3a,4,7,8,8a-hexahydro-7-methyl-3-methylene-	306	477303	1.71	C_17_H_22_O_5_
29.213	1,1’-Butadiynylenedicyclohexanol	246	1042563	3.75	C_16_H_22_O_2_
29.602	Spiro[tricyclo[4.4.0.0(5,9)]decane-10,2’-oxirane], 1-methyl-4-isopropyl-7,8-dihydroxy-, (8S)-	252	2196562	7.89	C_15_H_24_O_3_
30.013	6,9,12,15-Docosatetraenoic acid, methyl ester	346	658578	2.37	C_23_H_38_O_2_
30.549	2-[5-(2,2-Dimethyl-6-methylene-cyclohexyl)-3-methyl-pent-2-enyl]-[1,4]benzoquinone	312	1493226	5.37	C_21_H_28_O_2_
32.927	Butyl 4,7,10,13,16,19-docosahexaenoate	384	669066	2.40	C_26_H_40_O_2_
33.022	2(3H)-Naphthalenone, 4,4a,5,6,7,8-hexahydro-4a-phenyl-, (R)-	226	555633	2.00	C_16_H_18_O
33.622	2-Cyclohexene-1-carboxylic acid, 2-(7-hydroxy-3-methyl-1,3-octadienyl)-1,3-dimethyl-4-oxo-, methyl ester, [R-[R*,S*-(E,E)]]-	320	577481	2.07	C_19_H_28_O_4_
34.453	5-Benzofuranacetic acid, 6-ethenyl-2,4,5,6,7,7a-hexahydro-3,6-dimethyl-α-methylene-2-oxo-, methyl ester	276	1019099	3.66	C_16_H_20_O_4_
35.589	α-Santonin	246	377782	1.36	C_15_H_18_O_3_
36.452	Gibberellic acid	346	414448	1.49	C_19_H_22_O_6_
37.084	Azuleno[4,5-b]furan-2(3H)-one, decahydro-7,9-dihydroxy-6,9a-dimethyl-3-methylene-, [3aS-(3aα,6β,6aα,7α,9α,9aβ,9bα)]-	266	446152	1.60	C_15_H_22_O_4_
38.399	2-[4-methyl-6-(2,6,6-trimethylcyclohex-1-enyl)hexa-1,3,5-trienyl]cyclohex-1-en-1-carboxaldehyde	324	532212	1.91	C_23_H_32_O
38.525	1H-2,8a-Methanocyclopenta[a]cyclopropa[e]cyclodecen-11-one, 1a,2,5,5a,6,9,10,10a-octahydro-5,5a,6-trihydroxy-1,4-bis(hydroxymethyl)-1,7,9-trimethyl-, [1S-(1α,1aα,2α,5β,5aβ,6β,8aα,9α,10aα)]-	364	453515	1.63	C_20_H_28_O_6_
38.630	9,10-Secocholesta-5,7,10(19)-triene-1,3-diol, 25-[(trimethylsilyl)oxy]-, (3β,5Z,7E)-	488	278366	1.00	C_30_H_52_O_3_Si
39.840	2-[4-methyl-6-(2,6,6-trimethylcyclohex-1-enyl)hexa-1,3,5-trienyl]cyclohex-1-en-1-carboxaldehyde	324	351029	1.26	C_23_H_32_O
39.988	2H-Cycloheptafuran-2-one, 6-[1-(acetyloxy)-3-oxobutyl]-3,3a,4,7,8,8a-hexahydro-7-methyl-3-methylene-	306	313127	1.13	C_17_H_22_O_5_
40.787	Propanoic acid, 2-methyl-, (dodecahydro-6a-hydroxy-9a-methyl-3-methylene-2,9-dioxoazuleno[4,5-b]furan-6-yl)methyl ester, [3aS-(3aα,6β,6aα,9aβ,9bα)]-	350	815157	2.93	C_19_H_26_O_6_
41.545	2-[4-methyl-6-(2,6,6-trimethylcyclohex-1-enyl)hexa-1,3,5-trienyl]cyclohex-1-en-1-carboxaldehyde	324	692927	2.49	C_23_H_32_O

**Figure 1:**
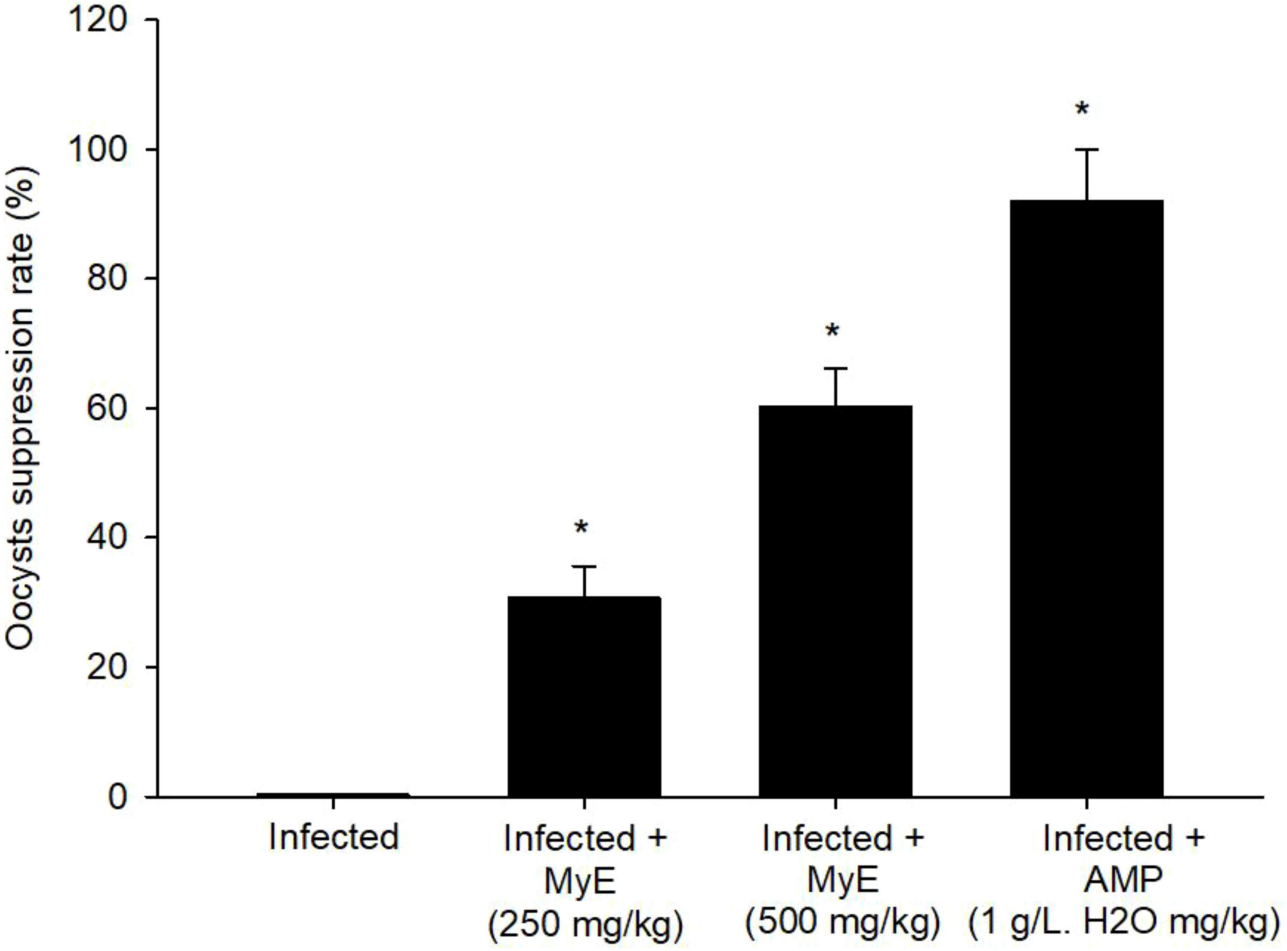
Suppression of *Eimeria labbeana*-like oocysts in infected and infected-treated pigeons. ^*^p ≤ 0.05, indicates a significant difference compared with the infected group. (MyE, myrrh extract; AMP, amprolium).

**Figure 2:**
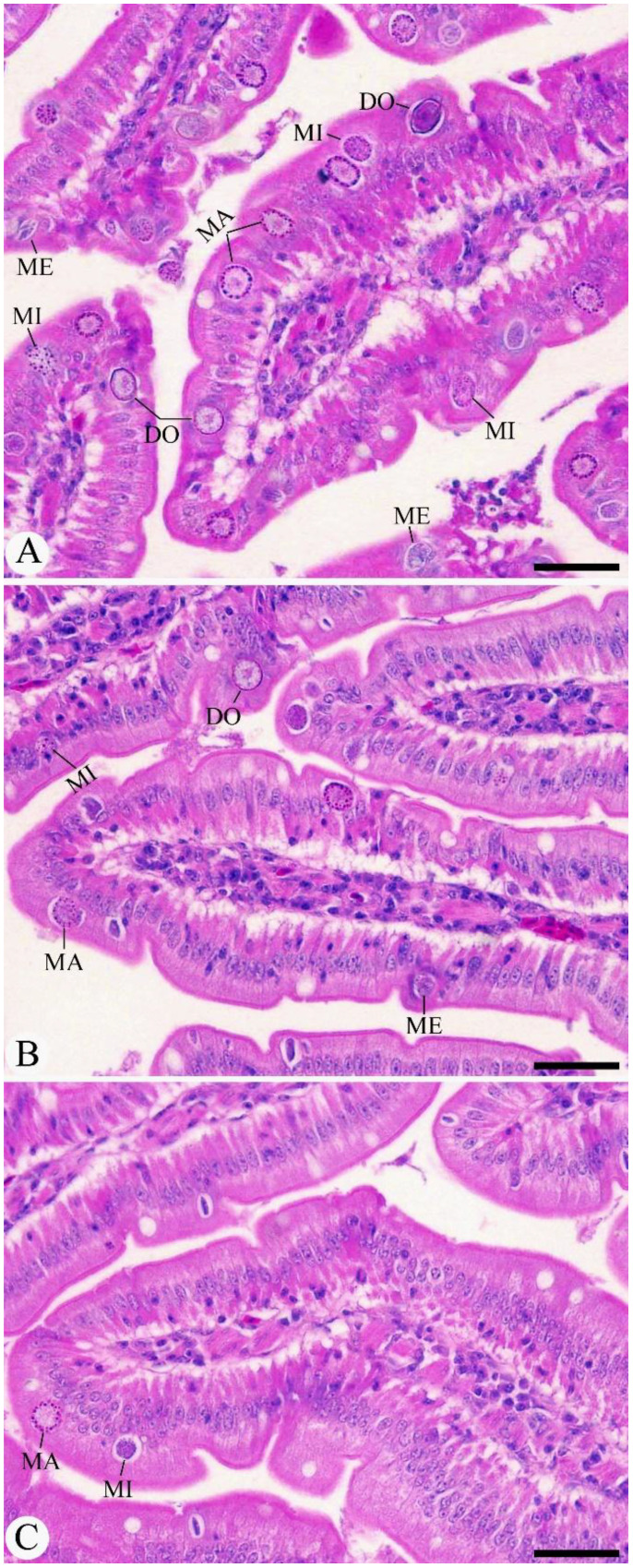
Histological sections of pigeon intestines across experimental groups. **(A)** Intestinal tissue from *E. labbeana*-like infected pigeons showing an elevated number of parasite stages. **(B, C)** Tissues from infected pigeons treated with MyE and AMP, respectively, showing reduced parasite stages. Scale bar = 50 µm (MyE, myrrh extract; AMP, amprolium).

**Figure 3:**
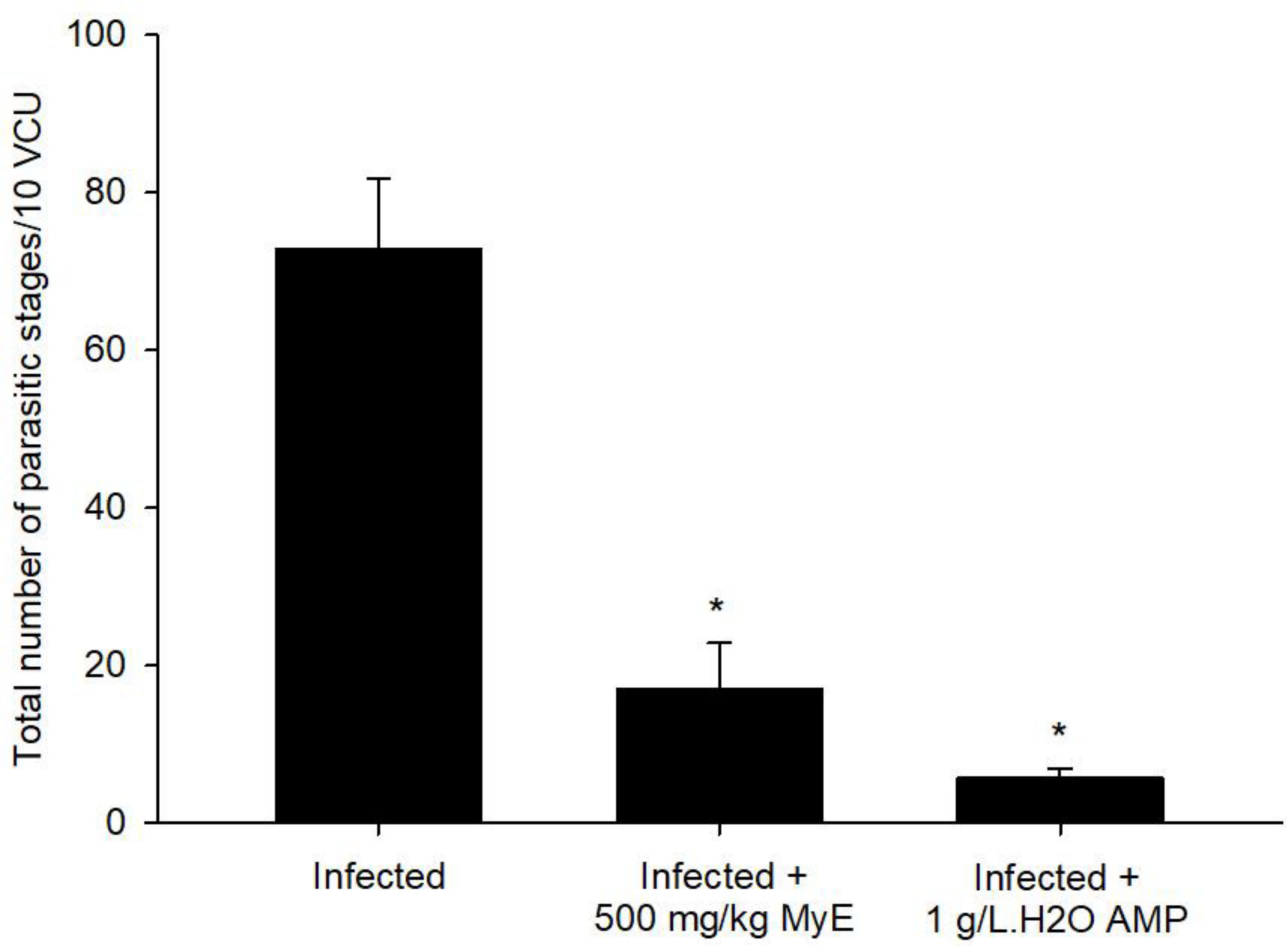
Effects of treatment with 500 mg/kg MyE and 1 g/L H_2_O AMP on the total number of *Eimeria labbeana*-like parasitic stages in the intestinal tissue per 10 VCU on day 8 p.i. ^*^p ≤ 0.05, indicates a significant difference compared with the infected group. (MyE, myrrh extract; AMP, amprolium).

On day 8 after infection, the peak shedding of fecal oocysts was observed in the infected, untreated group, reaching approximately 5.249 × 10^5^ ± 3.13 × 10^4^ oocysts per gram of feces. After treatment interventions, there was a significant reduction in oocyst output, with a 60.16% decrease compared to the infected group (**Figure 1**). Experimental infection of pigeons with *Eimeria* oocysts led to the development of various parasite stages (meronts, gamonts, and developing oocysts) in the intestinal epithelial cells, as seen in the H&E-stained sections (**Figure 2**). The number of intracellular parasitic stages was significantly lower in pigeons treated with MyE, decreasing from 72.91 ± 8.81 in the infected group to 16.99 ± 5.84 stages per 10 VCU, compared to the reference drug at 5.66 ± 1.15 stages per 10 VCU (**Figure 3**).

**Figure 4:**
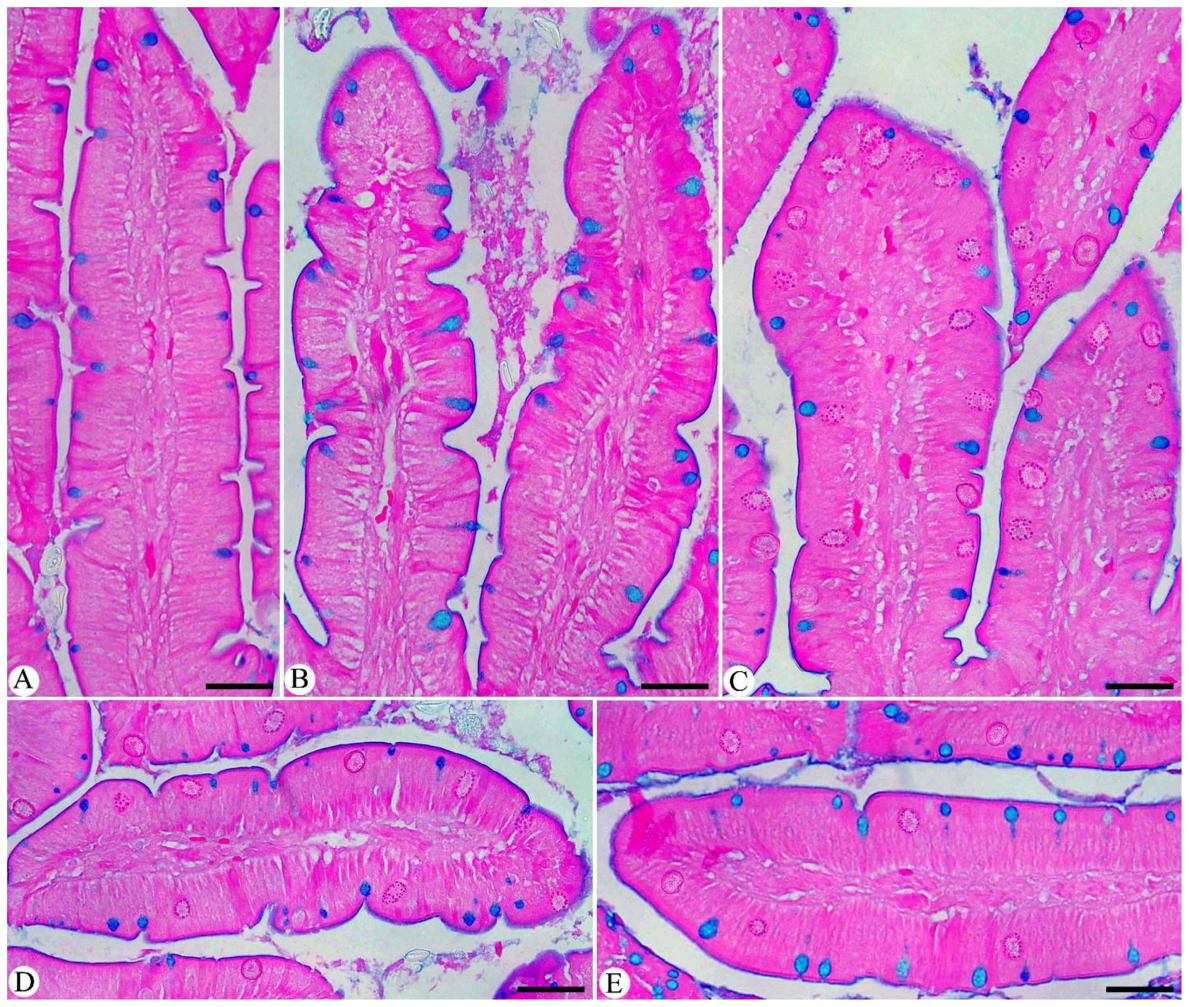
Small intestinal sections illustrating goblet cells in **(A)** control group, **(B)** non-infected pigeons treated with 500 mg/kg MyE, **(C)** infected group, **(D)** infected pigeons treated with 500 mg/kg MyE, and **(E)** infected pigeons treated with 1 g/L H_2_O AMP. Sections were stained with Alcian Blue and counterstained with eosin, and goblet cells were quantified in 10 well-oriented villus-crypt units (VCU). Scale bar = 100µm.

**Figure 5:**
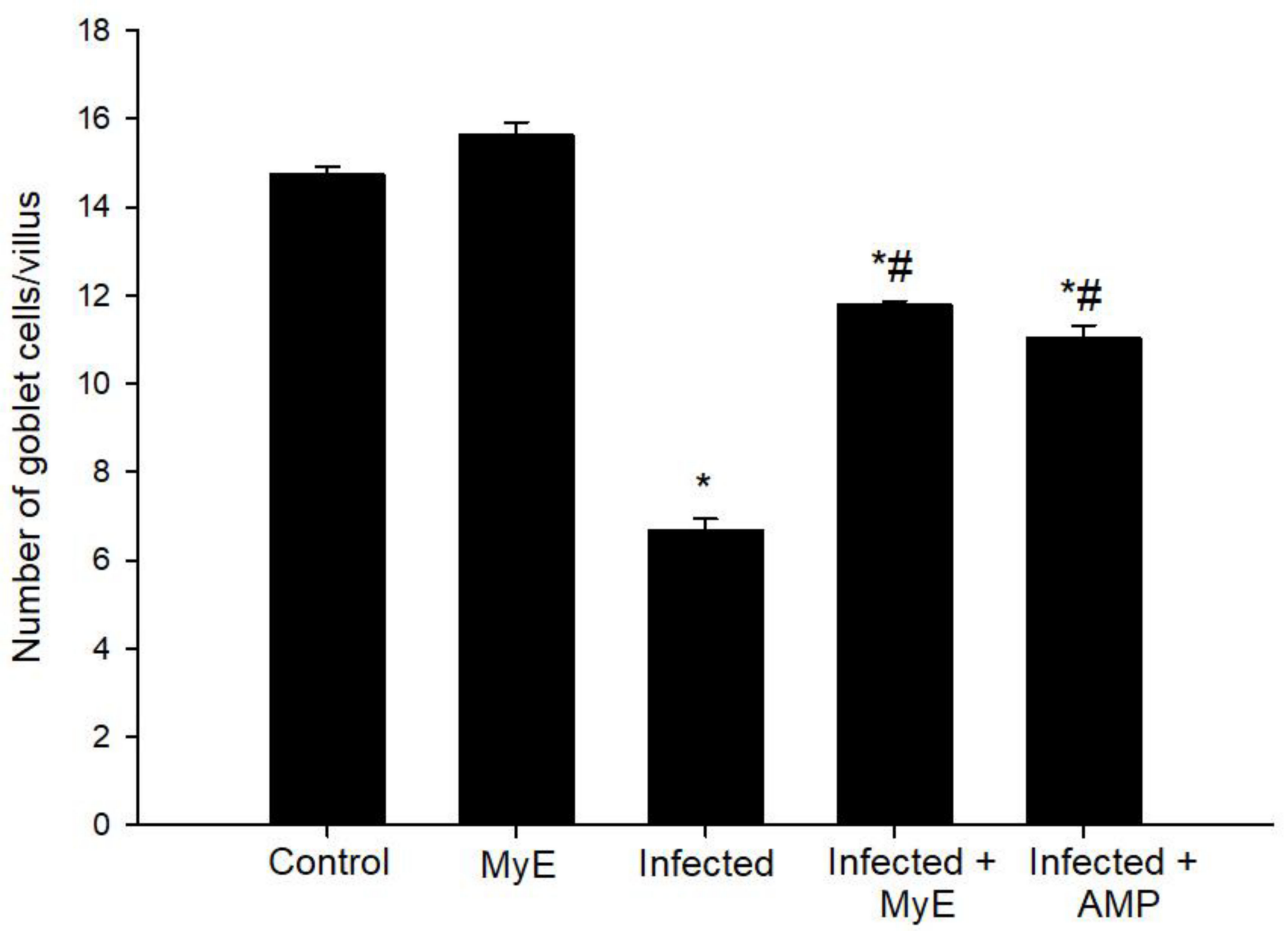
Variation in the number of intestinal goblet cells within villi among the control, non-infected treated with 500 mg/kgMyE, infected, and infected-treated groups (500 mg/kg MyE and 1 g/L H_2_O AMP). ^*^p ≤ 0.05, indicates a significant difference compared with the control group, ^#^p ≤ 0.05, indicates a significant difference compared with the infected group. (MyE, myrrh extract; AMP, amprolium).

Microscopic examination of intestinal sections stained with alcian blue (**Figure 4**) showed significant histological changes after pigeons were infected with *Eimeria*. Specifically, there was a marked decrease in goblet cell numbers, with an average of 6.69 ± 0.25 goblet cells per VCU in the infected group. In contrast, the control group had a higher average of 14.74 ± 0.18 goblet cells/VCU (**Figures 4**, **5**). When infected pigeons were treated with MyE, the number of goblet cells increased significantly, reaching an average of 11.79 ± 0.09 goblet cells/VCU.

**Figure 6:**
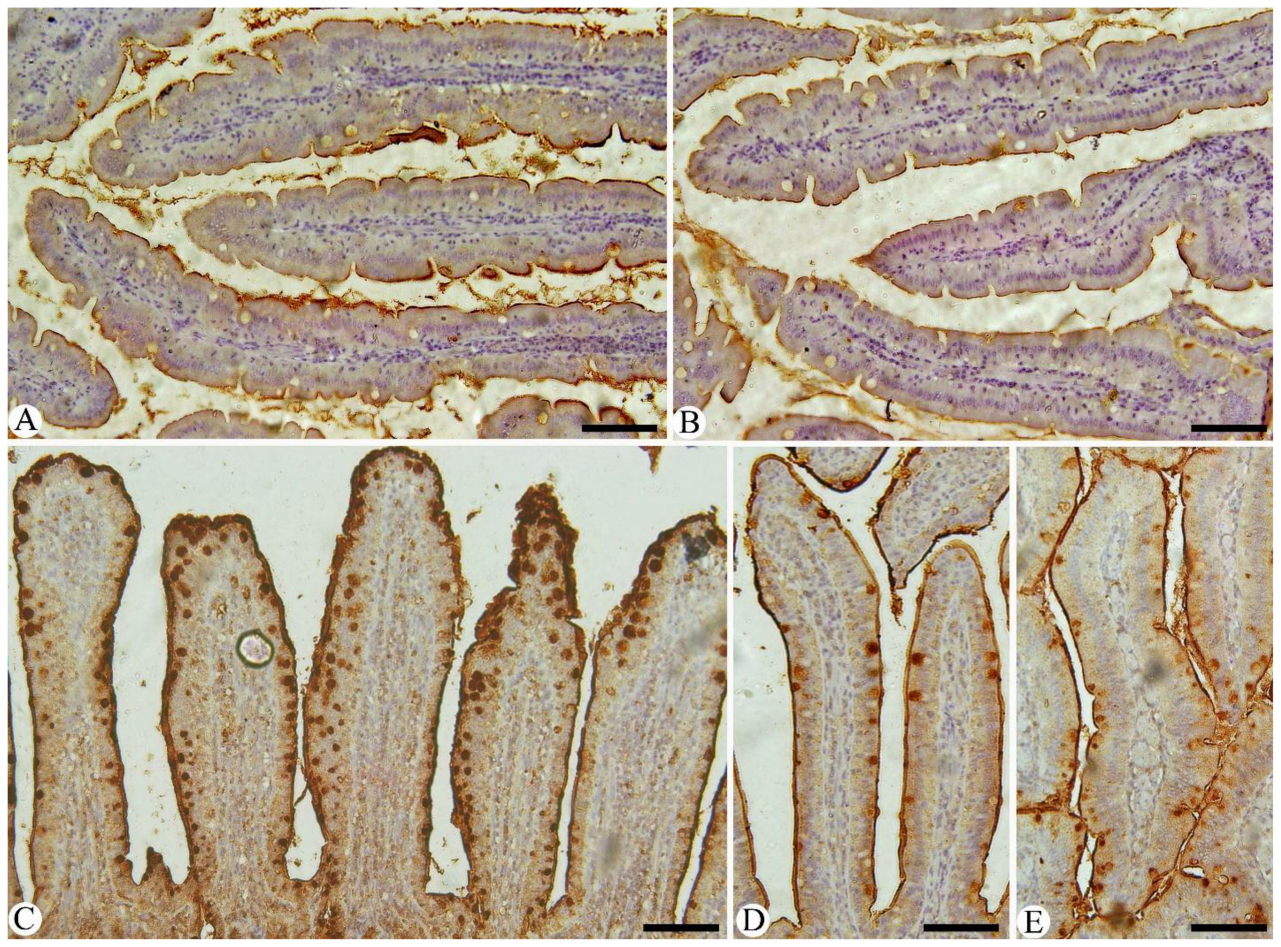
Immunohistochemical detection of NF-KB in the intestinal tissues of pigeons. **(A)** control group, **(B)** non-infected group treated with 500 mg/kg MyE, **(C)** infected group, **(D)** infected group treated with 500 mg/kg MyE, and **(E)** infected group treated with amprolium. Scale Bar = 100 µm.

**Figure 7:**
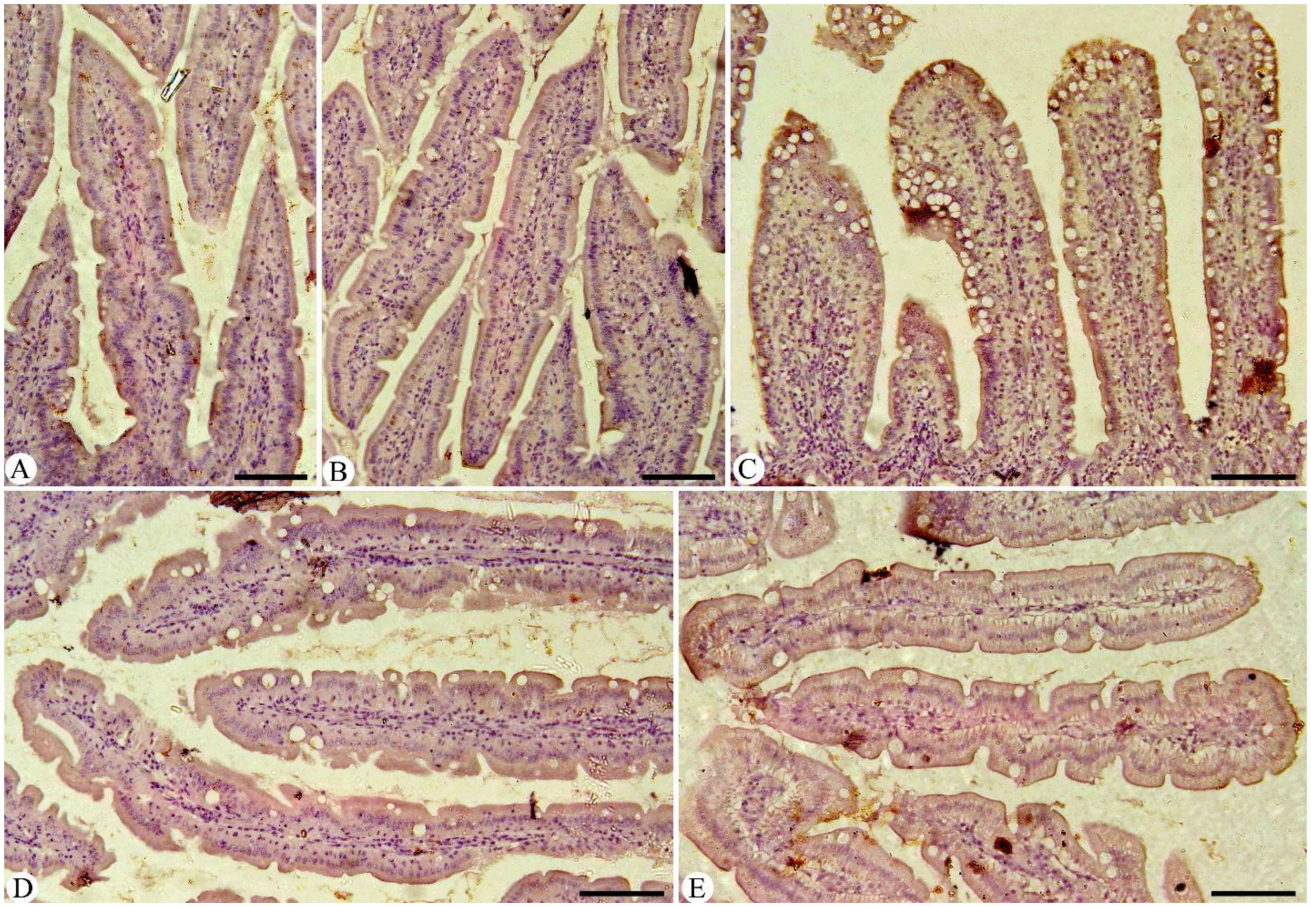
Immunohistochemical detection of IFN-γ in intestinal tissues of pigeons. **(A)** Control pigeon group, **(B)** non-infected group treated with 500 mg/kg MyE, **(C)** infected group, **(D)** infected group treated with 500 mg/kg MyE, and **(E)** infected group treated with amprolium. Scale Bar = 100 µm.

Intestinal sections from various experimental groups underwent immunohistochemical staining specifically to identify and quantify NF-kB (**Figure 6**) and IFN-γ (**Figure 7**) positive cells. In the control group, the number of positive cells was within normal physiological levels. However, the results showed that *Eimeria* infection caused a strong immunoreactivity to NF-kB and IFN-γ-positive intestinal cells compared to the control pigeons. After administering MyE, a significant decrease in immunoreactivity to NF-kB and IFN-γ in the intestinal tissues was observed in the intestinal sections of pigeons affected by *Eimeria* infections.

**Figure 8:**
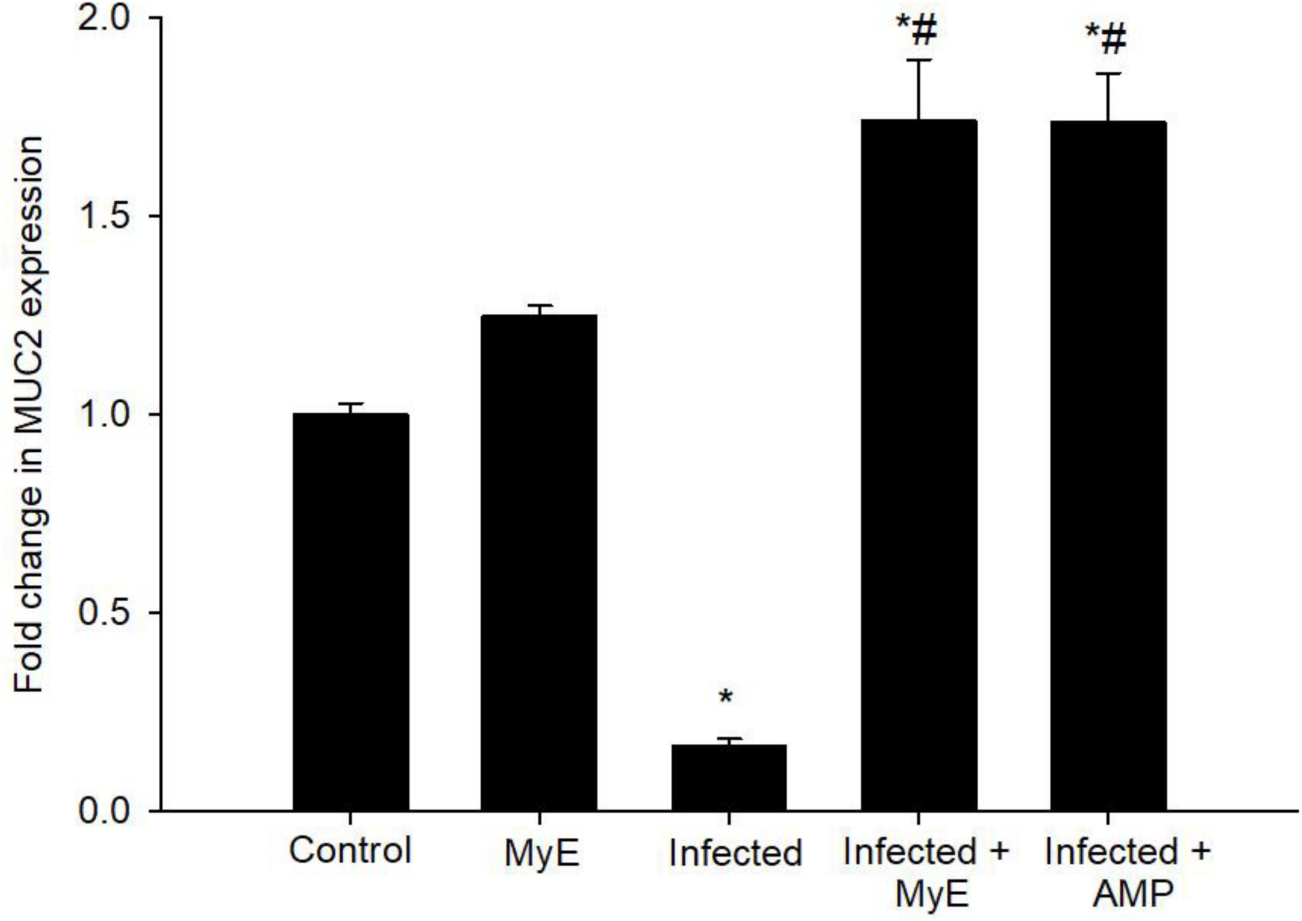
Effect of MyE treatment on *MUC2* mRNA expression in intestinal tissues from *E. labbeana*-like infected pigeon. Expression values determined by RT-PCR were normalized to β-actin and presented as fold change (log 2 scale) relative to the control group. ^*^p ≤ 0.05, indicates a significant difference compared with the control group, ^#^p ≤ 0.05, indicates a significant difference compared with the infected group. (MyE, myrrh extract; AMP, amprolium).

**Figure 9:**
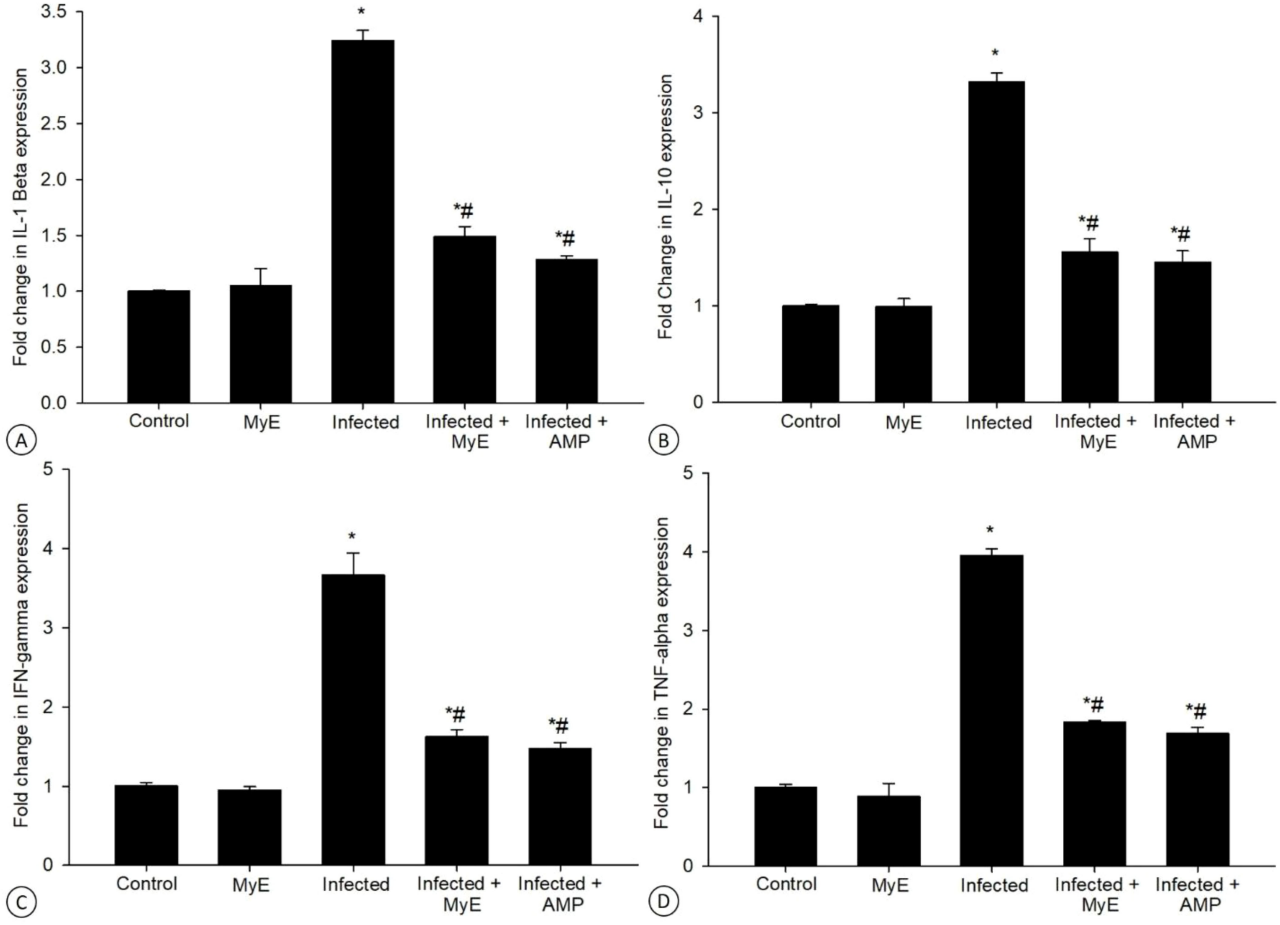
Effect of MyE treatment on the mRNA expression of **(A)** IL-1β, **(B)** IL-10, **(C)** IFN-γ, and **(D)** TNF-α in intestinal tissues of *E. labbeana*-like infected pigeons. Expression levels determined by RT-PCR were normalized to β-actin and expressed as fold change (log 2 scale) relative to the control group. ^*^p ≤ 0.05, indicates a significant difference compared with the control group, ^#^p ≤ 0.05, indicates a significant difference compared with the infected group. (MyE, myrrh extract; AMP, amprolium).

qRT-PCR was used to measure the mRNA levels of inflammatory cytokines in pigeon intestinal tissues (**Figures 8**, **9**). A notable decrease in MUC2 gene expression was seen after *Eimeria* infection. Treatment with MyE significantly boosted MUC2 expression, increasing from 0.16 to 1.74-fold (**Figure 8**).

Following the *Eimeria* infection, there was a significant increase in the mRNA expression of the IL-1β gene. This expression increased approximately 3.24 times compared to the control group (**Figure 9A**). Additionally, treatment with MyE caused a notable decrease in this gene’s expression, roughly 1.48 times lower than in the infected group. In comparison, the reference drug resulted in a 1.28-fold reduction in expression (**Figure 9A**).

The infection caused by *Eimeria* significantly increased the mRNA expression levels of the IL-10 gene. Specifically, this increase was approximately 3.31 times higher than in the control group (**Figure 9B**). In contrast, the administration of MyE resulted in a notable decrease in IL-10 expression, with levels dropping about 1.55-fold compared to the reference drug (1.45-fold).

*Eimeria* infection leads to a significant increase in the mRNA expression of the IFN-γ gene. This upregulation is approximately 3.66 times higher than in the control group (**Figure 9C**). Treatment with MyE causes a notable reduction in IFN-γ gene expression, bringing it down to about 1.62 times that of the control group. This decrease is less pronounced than with the reference drug, which shows a 1.47-fold change (**Figure 9C**).

Following infection with the *Eimeria* parasite, a significant increase in the mRNA expression levels of the TNF-α gene was observed. This increase was approximately 3.95 times higher than the control group, which had a baseline expression level of 1.00-fold (**Figure 9D**). After treatment with MyE, a notable reduction in TNF-α mRNA expression occurred, bringing it to about 1.83-fold. This is a decrease compared to the reference drug, which had a 1.69-fold expression level.

**Figure 10:**
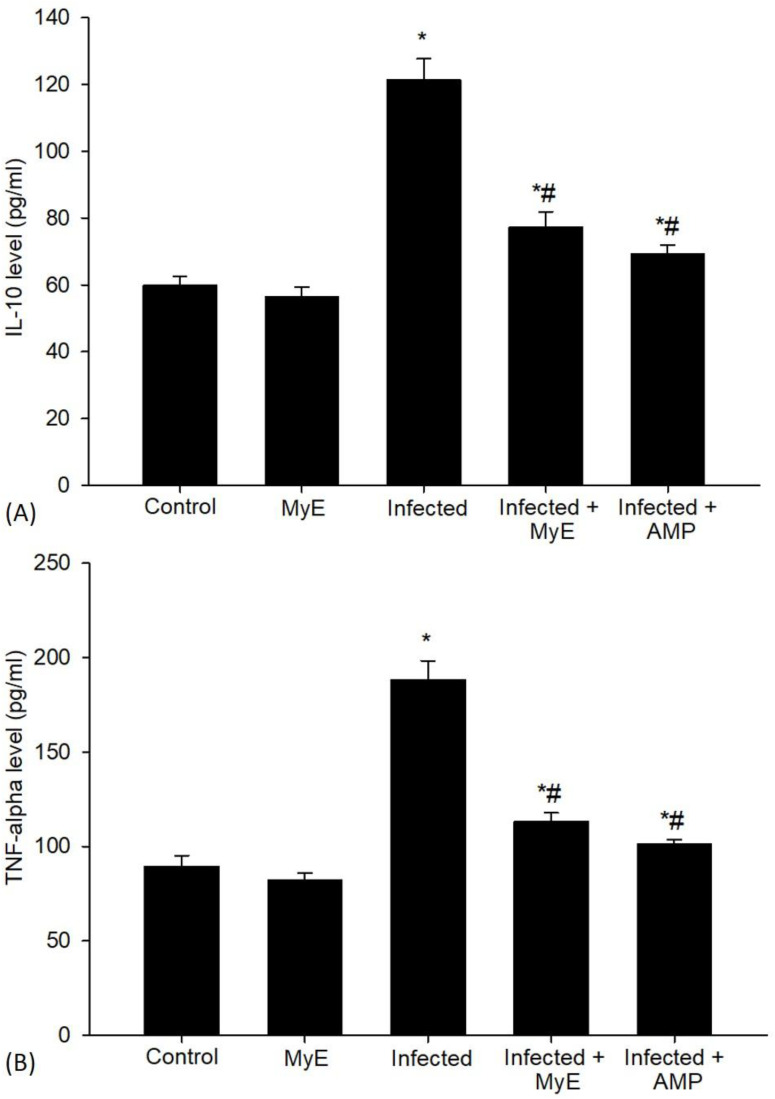
Levels of **(A)** IL-10 and **(B)** TNF-α in intestinal tissues of pigeons across different experimental groups. ^*^p ≤ 0.05, indicates a significant difference compared with the control group, ^#^p ≤ 0.05, indicates a significant difference compared with the infected group. (MyE, myrrh extract; AMP, amprolium).

To measure the levels of the anti-inflammatory cytokine IL-10, we used ELISA (**Figure 10A**). The results showed that *Eimeria* infection caused a significant increase in IL-10 levels, reaching 121.22 ± 6.46 pg/ml. This was much higher than the baseline levels seen in the control group, which averaged 59.84 ± 2.74 pg/ml. In contrast, MyE treatment significantly reduced the elevated IL-10 levels caused by *Eimeria* infection, decreasing the cytokine level to 77.21 ± 4.63 pg/ml.

Following infection with *Eimeria*, TNF-α levels rose significantly, reaching an average of 188.23 ± 10.01 pg/ml (**Figure 10B**). This sharp increase was notably higher than in the control group, which had an average of 89.22 ± 5.95 pg/ml. The infected pigeons treated with MyE showed a significant decrease, with TNF-α levels dropping to an average of 113.07 ± 4.68 pg/ml.

## Discussion

Coccidiosis is a protozoan disease that affects the intestines of various animal species and poses a significant economic concern in poultry production. Although several anticoccidial drugs are widely used to control the disease (33), the emergence of drug-resistant *Eimeria* strains has prompted the exploration of alternative strategies, including plant-derived anticoccidial agents (34). In poultry, different plant extracts have been shown to reduce *Eimeria* replication, enhance intestinal integrity, and lessen disease severity (35, 36). Building on these findings, the present study evaluated the effectiveness of *Commiphora myrrha* resin in managing *E. labbeana*-like infections in pigeons, using a high dose of myrrh extract (500 mg/kg) as a natural therapeutic approach to reduce inflammation and intestinal damage associated with infection.

The results demonstrated that experimental infection with *E. labbeana*-like oocysts followed by MyE treatment significantly decreased oocyst output compared with the infected control group. This reduction may be attributed to the high phenolic content of MyE, which exerts multiple biological effects (37, 38). Similar outcomes have been reported in poultry coccidiosis, where phenolic-rich plant extracts substantially reduced oocyst shedding, inhibited sporulation, disrupted oocyst wall integrity, and enhanced host resistance to *Eimeria* infections (39–41). (1) directly disrupt parasite structures—Phenolics and related bioactive molecules can interact with the lipid and protein components of sporozoite and oocyst membranes, causing structural destabilization, impaired sporulation, and lowered infectivity. (2) Provide antioxidant-mediated mucosal protection—By scavenging ROS produced during infection, MyE guards intestinal epithelial cells from oxidative damage, maintaining barrier integrity and reducing parasite adhesion and invasion. (3) Modulate host immune responses—Phenolic compounds can boost both innate and adaptive immunity, increasing cytokines such as IFN-γ and IL-10, activating macrophages, and promoting mucin secretion by goblet cells, collectively hindering *Eimeria* colonization.

Histopathological examination revealed severe intestinal lesions and multiple developmental stages of *Eimeria* in infected pigeons, similar to observations in poultry infected with *Eimeria* species, which show epithelial damage, villous atrophy, and mucosal disruption (36, 42–45). In line with studies on *E. tenella* infections in chickens (46), untreated pigeons exhibited extensive tissue pathology, whereas treatment with MyE significantly reduced parasite developmental stages and mitigated intestinal lesions. Mechanistically, bioactive compounds in MyE likely interfere with parasite invasion, replication, and intracellular development by disrupting parasite enzyme activity and organelle function. Additionally, the antioxidant and phenolic constituents of MyE may further suppress coccidian proliferation by inducing oxidative stress within the parasite while neutralizing ROS in host tissues, consistent with mechanisms described in poultry coccidiosis research (23). These findings underscore the complex interplay between *Eimeria* developmental stages and host-pathogen interactions, highlighting the potential of MyE as a therapeutic agent in managing coccidiosis in pigeons.

One of the key cellular immune responses in the avian intestine is the goblet cell response, which helps form the protective mucosal barrier. During *Eimeria* infection, a widespread decrease in goblet cell numbers has been reported in chickens and other birds, leading to less mucin secretion and higher vulnerability to intestinal damage (47). This decrease is mainly caused by parasite-induced epithelial damage and the local inflammatory response, which trigger apoptosis of goblet cells and hinder the differentiation of intestinal stem cells into mucin-producing cells (48). In this study, treatment with *C. myrrha* extract (MyE) restored goblet cell numbers in infected pigeons. Mechanistically, MyE appears to directly protect the intestinal epithelium by reducing oxidative stress and modulating inflammatory signaling pathways such as NF-κB and MAPK, which in turn promote goblet cell survival and differentiation. This restoration likely enhanced the mucosal barrier, increased mucin secretion, and decreased the parasite’s ability to invade or multiply within the intestinal epithelium, leading to a reduction in fecal oocyst shedding. Similar protective effects on goblet cells and mucosal integrity have been seen in poultry coccidiosis following plant-based treatments (34, 36). For example, supplementing with ginger extract has been shown to lower fecal oocyst counts and improve histopathological changes in the cecum of infected birds, possibly through anti-inflammatory and antioxidant mechanisms that help preserve epithelial integrity (49). Additionally, studies show that plant compounds from *Rumex nervosus* and *Cassia alata* can boost goblet cell activity and mucin production by upregulating transcription factors like SPDEF and KLF4, which drive goblet cell differentiation, thereby strengthening the intestinal barrier during *Eimeria* infections (50, 51). The results of this study indicated that *Eimeria* infection caused a decrease in the goblet cell MUC2 gene, which is mainly expressed in small intestinal goblet cells. This finding aligns with earlier studies in poultry, where *Eimeria* infections (such as *E. tenella* and *E. maxima*) reduced MUC2 expression and goblet cell numbers, weakening the mucosal barrier and increasing vulnerability to intestinal damage (48, 52). The downregulation of MUC2 during infection is driven by parasite-induced epithelial stress, pro-inflammatory cytokines (as TNF-α and IFN-γ), and oxidative damage, which impair goblet cell function and mucin gene transcription. The MUC2 gene plays a crucial role in innate intestinal defense by regulating mucin secretion and modulating immune and inflammatory responses against pathogen-induced injury. In this study, MyE treatment suppressed *Eimeria* development in the intestine and simultaneously activated signaling pathways that increase MUC2 transcription, such as the EGFR-MAPK and Notch pathways, leading to higher goblet cell numbers and more mucin secretion. This dual effect not only strengthened mucosal protection but also improved the host’s inflammatory response, consistent with earlier coccidiosis studies (15, 53). Recent studies have further clarified MUC2’s role in poultry gut health. For example, El-Sayed et al. (54) showed that *Holothuria polii* extract (HpE) significantly reduced oocyst output and increased MUC2 gene expression in infected mice, suggesting its potential as an anticoccidial agent through strengthening the mucosal barrier and influencing local cytokine production. Similarly, research on *Rumex nervosus* leaf extracts found that it boosts goblet cell regulation and the inflammatory response during *E. tenella* infection in chickens by lowering pro-inflammatory cytokine signals and promoting mucin gene expression, highlighting the therapeutic potential of plant-based treatments (55). Furthermore, Elshershaby et al. (56) reported that *Cassia alata* extract increased goblet cell numbers and lowered macrophage infiltration in the intestinal villi of infected chickens, supporting the benefits of natural extracts in improving gut health by reducing excessive inflammation and maintaining epithelial integrity. These findings emphasize the importance of maintaining goblet cell function and MUC2 expression to fight *Eimeria* infections. Enhancing MUC2 levels through treatments like MyE and natural extracts not only boosts the intestinal barrier but also modulates the mucosal immune response, including balancing pro- and anti-inflammatory cytokines, offering promising strategies for improving poultry health and reducing reliance on conventional anticoccidial drugs.

Moreover, this study demonstrated that pigeons inoculated with *E. labbeana*-like oocysts exhibited upregulation of pro-inflammatory cytokines (IL-1β, IFN-γ, and TNF-α), anti-inflammatory cytokine (IL-10), and transcription factor (NF-κB), which activates the expression of pro-inflammatory genes such as IL-1β and TNF-α. Together, these molecules play critical roles in immune responses and the development of various inflammatory diseases. This upregulation was associated with the generation of reactive oxygen species (ROS), which in turn elevated NO and iNOS levels, contributing to oxidative stress that can damage both parasite and host tissues. The induction of these markers is likely mediated through pattern recognition receptors (PRRs), such as Toll-like receptors (TLRs), which detect parasite antigens and activate downstream signaling pathways, including NF-κB and MAPK pathways, resulting in transcription of pro-inflammatory cytokines and chemokines. These responses corresponded with oocyst shedding in the experimental group, reflecting the host’s attempt to limit parasite proliferation. These results align with those of Laurent et al. (57), who found that a single *E. tenella* infection triggered a host immune response with significant expression of IL-10 and IFN-γ in the ceca. Similarly, Hong et al. (58) reported marked increases in IL-10 and IFN-γ mRNA in CD4^+^ T cells of chickens infected with *E. maxima*, highlighting the role of adaptive Th1/Th2 responses. Lillehoj and Trout (59) also noted that proinflammatory cytokines such as IL-1β and TNF-α are upregulated early in *Eimeria* infection, functioning to recruit macrophages, neutrophils, and NK cells to infected sites and promote phagocytosis of sporozoites and merozoites. Yun et al. (6) observed that *E. acervulina* infection significantly elevated IFN-γ and IL-10 in intestinal lymphocytes, emphasizing their role in balancing Th1-mediated protection and immunopathology. More recently, Kim et al. (60) confirmed that NF-κB activation is central to initiating transcription of inflammatory mediators during poultry coccidiosis. In line with Bremner et al. (61), serum IL-10 levels were elevated following *E. maxima* infection, suggesting an anti-inflammatory feedback mechanism to prevent excessive tissue damage. Chow et al. (62) reported that the expression of NF-κB and IFN-γ—a key cytokine driving cell-mediated immunity—is influenced by IL-10 induction. IFN-γ, produced by NK and T cells, activates macrophage cytotoxic functions, enhances antigen presentation, and promotes the migration of neutrophils and macrophages to infection sites, where they target intracellular *Eimeria* stages. These observations are consistent with reports showing a pronounced intestinal IFN-γ response during infections with *E. maxima* (63), *E. bovis* and *E. alabamensis* (64), and *E. tenella* (6). Recent studies indicate that myrrh extract (MyE) not only targets intracellular *Eimeria* parasite stages but also acts as an immunomodulatory agent, protecting host tissues. Mechanistically, MyE components, particularly flavonoids and terpenoids, are hypothesized to inhibit NF-κB nuclear translocation, suppress ROS production, and potentially modulate signaling pathways such as MAPK and JAK/STAT, thereby contributing to the downregulation of proinflammatory cytokines. These proposed mechanisms remain to be directly confirmed in future experiments. Specifically, MyE has been shown to reduce fecal oocyst shedding and impair *Eimeria* parasite development and maturation in infected pigeons. Consistent with poultry coccidiosis studies of Lillehoj and Lillehoj (65) and Chapman et al. (5), MyE attenuated the inflammatory response by significantly downregulating the expression of selected inflammatory markers in the intestinal tissue. This decline highlights the anti-inflammatory and immunomodulatory roles of flavonoids, attributed to their hydroxyl groups, which help modulate cytokine secretion, suppress ROS-induced tissue injury, and maintain Th1/Th2 immune balance in hosts infected with *Eimeria* (66–68). Similar mechanisms have been described in poultry studies of Al-Samarrai et al. (37) Abbas et al. (40), and El-Shall et al. (13), where flavonoid-rich extracts decreased pro-inflammatory cytokines, modulated T-cell responses, and reduced intestinal lesions caused by *Eimeria* species. Although the current findings support the therapeutic potential of *C. myrrha* extract, it is important to acknowledge that this study did not evaluate the acute or sub-chronic toxicity or overall safety profile of MyE. The absence of toxicity assessment limits definitive conclusions regarding its safety for long-term therapeutic or prophylactic use. Therefore, future studies should incorporate comprehensive toxicity testing, dose–response evaluations, and pharmacokinetic analyses to establish safe and effective administration guidelines.

## Conclusion

The findings of this study demonstrate that *C. myrrha* extract provides significant protective effects against *E. labbeana*-like–induced intestinal damage and inflammatory dysregulation in pigeons. The extract’s bioactive constituents appear to modulate pro- and anti-inflammatory pathways, mitigate oxidative stress, and preserve intestinal integrity, ultimately reducing oocyst shedding and improving histological outcomes. These results highlight the potential of *C. myrrha* as a natural alternative for managing avian coccidiosis. However, important limitations should be acknowledged. The small sample size, absence of independent experimental replication, and lack of acute or sub-chronic toxicity assessment restrict the generalizability, clinical relevance, and safety-related conclusions of this work. Without formal toxicity evaluation, definitive statements regarding the extract’s suitability for therapeutic or prophylactic use remain premature. Therefore, future studies using larger cohorts, replicated trials, dose–response analyses, and comprehensive safety assessments are essential to validate and extend these promising findings.

## Data Availability

The original contributions presented in the study are included in the article/supplementary material. Further inquiries can be directed to the corresponding author.
